# A note on internet use and the 2016 U.S. presidential election outcome

**DOI:** 10.1371/journal.pone.0199571

**Published:** 2018-07-18

**Authors:** Levi Boxell, Matthew Gentzkow, Jesse M. Shapiro

**Affiliations:** 1 Economics Department, Stanford University, Stanford, CA, United States of America; 2 National Bureau of Economic Research, Cambridge, MA, United States of America; 3 Economics Department, Brown University, Providence, RI, United States of America; University of Toronto, Rotman School, CANADA

## Abstract

We use data from the American National Election Studies from 1996 to 2016 to study the role of the internet in the 2016 U.S. presidential election outcome. We compare trends in the Republican share of the vote between likely and unlikely internet users, and between actual internet users and non-users. Relative to prior years, the Republican share of the vote in 2016 was as high or higher among the groups least active online.

## Introduction

Many have hypothesized that the internet and social media impacted the outcome of the 2016 U.S. presidential election. In a post-election interview, Hillary Clinton emphasized the role of social media in the election, citing fake news, Russian intervention, and Republicans’ success in “marrying content with delivery and data” [1]. Others have emphasized the Trump campaign’s use of data to target messages online [2].

There have been several attempts at examining these claims about the 2016 election empirically. Some argue that the internet is unlikely to have helped Trump because only a small percentage of Trump supporters use social media and because Trump did unusually well among the demographic groups least likely to use the internet [3]. Others show that while fake news was dominantly pro-Trump, it would have to be extraordinarily persuasive relative to other media technologies (e.g., TV ads) in order for it to have swayed the election [4].

We use data from the American National Election Studies (ANES) from 1996 to 2016 to study the role of the internet in the 2016 election outcome. Following closely the methodology used in a prior study of political polarization [5], we compare trends in the Republican share of the vote between likely and unlikely internet users, and between actual internet users and non-users. Relative to prior years, the Republican share of the vote in 2016 was as high or higher among the groups least active online.

Under the assumptions that (i) the internet affects elections only by changing the partisan vote share among those active on the internet, (ii) the effects of the internet on voting behavior are identical across individuals, and (iii) no other time-varying factors affected the difference in Republican vote share between internet-active and internet-inactive groups, our findings imply that the internet was not a source of advantage to Trump. (See Model section of the S1 Appendix).

Alternatively, our findings may be viewed as implying that, if the internet was a significant source of advantage to Trump, at least one of assumptions (i), (ii), or (iii) must be violated in a quantitatively significant way. We discuss this possibility in more detail in the concluding section.

## Data

We use data from the ANES [6–9], which is a nationally representative survey that asks various demographic and political questions. We use the ANES 1948–2012 Time Series Cumulative, 2008 Time Series, 2012 Times Series, and 2016 Time Series datasets. We use data from survey waves in presidential election years from 1996–2016, inclusive, and we restrict attention to face-to-face surveys, excluding internet-based surveys that were conducted in more recent years. Our calculations weight responses from 1996–2012 by the type-0, face-to-face survey weights and responses from 2016 by the post-election, face-to-face survey weights.

Our outcome variable is the party that the respondent voted for in the most recent presidential election. We construct this variable from responses to “How about the election for President? Did you vote for a candidate for President? (IF YES:) Who did you vote for?” which are then coded as either Republican, Democratic, Other, or refusals for respondents who said they voted for a presidential candidate. Respondents who report not voting for a presidential candidate or who refuse to say who they voted for are excluded from our main analysis.

We use three different measures of internet use. Our first measure, which we refer to as whether or not a respondent uses the internet, comes from responses to “Do you have access to the Internet or the World Wide Web [exc. 2008: (‘the Web’)]?” for 1996–2008 and “Do you or anyone in this household use the Internet at any location?” for 2012–2016. Our second measure, which we refer to as whether or not a respondent observed campaign news online, comes from responses to “Have you seen any information about this election campaign on (the Internet/the Web)?” for 1996–2004, “Did you read, watch, or listen to any information about the campaign for President on the Internet?” for 2008–2012, and whether respondents “heard anything about the presidential campaign” on “Internet sites, chat rooms, or blogs” for 2016. Our third measure, which we refer to as predicted internet access, comes from [5] and classifies respondents according to whether the respondent is in the top or bottom quartile in terms of the likelihood of having internet if they were a respondent in 1996, as predicted from the following covariates: age group, gender, race, education, and whether the respondent lives in the political south. Table 1 shows the regression used to construct the predicted internet measure.

**Table 1:** Predicted internet, 1996.

	Estimator: Weighted least squares Dependent variable: Internet use
Intercept	0.420 (0.043)
Age Group: 40-64	-0.124 (0.022)
Age Group: 65+	-0.269 (0.030)
Gender: Male	0.008 (0.020)
Race: Hispanic	0.046 (0.045)
Race: Other	0.108 (0.069)
Race: White	0.156 (0.034)
Education: Grade School	-0.363 (0.053)
Education: High School	-0.371 (0.026)
Education: Some College	-0.146 (0.029)
Region: South	0.081 (0.022)
N	1513
*R*^2^	0.220

Notes: Table comes from the SI Appendix of [5]. Table shows the coefficients from a weighted least squares regression. Weights are the ANES survey weights. For estimation, the sample is restricted to respondents in 1996 who have valid responses to the questions needed to construct each independent and dependent variable. Dependent variable is an indicator for whether an individual uses the internet taken from the ANES (see SI appendix of [5] for details on the variable construction). All covariates are indicator variables. Conventional standard errors are in parentheses.

Separately for each measure of internet use, we exclude respondents with missing or non-valid responses (as defined by the ANES) to the questions needed to construct the measure. For the predicted internet measure in 2016, we also drop respondents whose response for education is in the “95. Other SPECIFY” category. Please see S1 Replication Code for exact details on the variables and samples used along with their construction.

## Results

**Fig 1:**
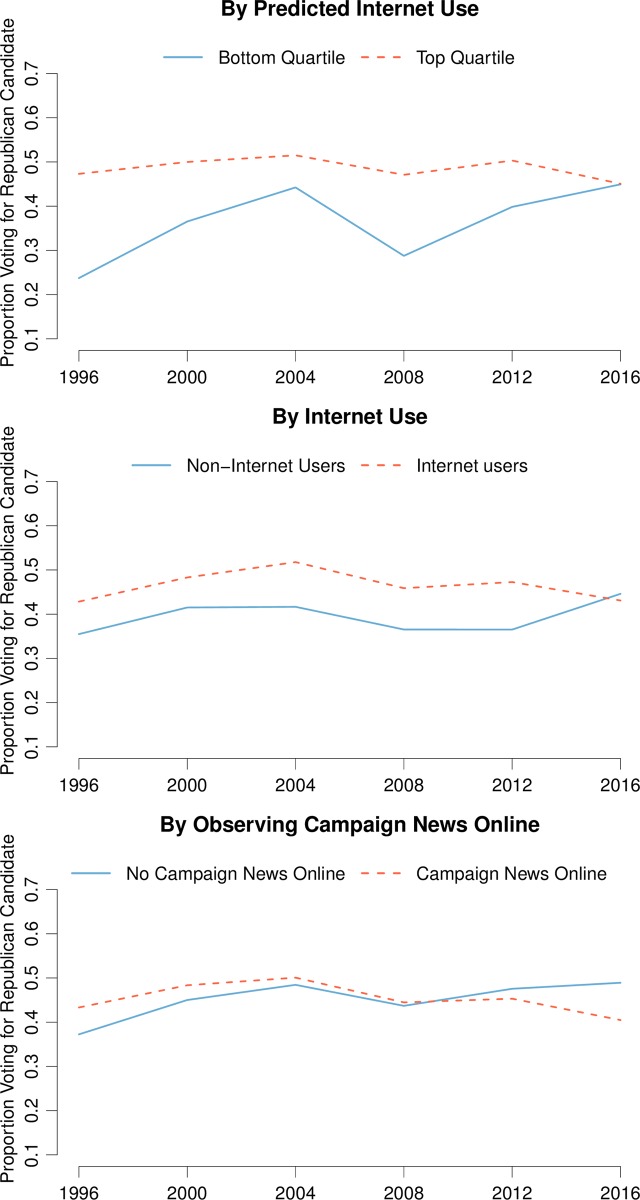
Trends in votes for Republican presidential candidate by online activity. Notes: Plot shows trends in the weighted proportion of voting respondents that voted for the Republican presidential candidate, separately for groups that are more and less active online. We measure online activity using predicted internet use, actual internet use, and whether or not the respondent observed campaign news online. See main text for details on variable construction.

Fig 1 shows, for each of our three measures of internet use, the proportion of voting respondents who voted for the Republican candidate in each presidential election. All three plots show that, if anything, Trump outperformed relative to trend among those groups that are least active online. For two of the three measures, the 2016 election marked the first time since 1996 that the Republican candidate performed equally well or better among the group that is less active online.

It is important to note that the composition of internet users is changing over time. Therefore, trends in, say, the Republican share among actual internet users reflect changes in respondents’ likelihood of internet use and changes in respondents’ voting behavior. Our measure of predicted internet use is constructed from a time-invariant function of covariates and is therefore less subject to this caveat.

It is also important to note that some respondents do not report a vote. The S1 Appendix reports trends in the proportion of respondents who do not report a vote, separately for groups with high and low internet use. In some cases the trends differ between the groups. If these trends are driven by survey nonresponse, and if nonresponse differs between Republican and Democrat voters, then this could be a source of bias in our analysis.

Table 2 shows, for each of our three measures of internet use, the change in the proportion of voting respondents who voted for the Republican candidate between 2012 and 2016, separately for more and less internet-active groups. The table also shows the difference in change in proportions between more and less internet-active groups. We report a 95 percent confidence interval on the change in proportions, and on the difference in change in proportions, based on a nonparametric bootstrap with 100 replicates. We find that, compared to Romney, Trump performed relatively better among less internet-active groups, though we note that the confidence intervals are wide and always include 0. Table 3 reports the sensitivity of our findings to changes in the covariate set used to construct the predicted internet use measure.

**Table 2:** Votes for Republican presidential candidate by online activity, 2012–2016.

Demographic group	Change in proportion	95% CI
By Predicted Internet Use:		
Bottom Quartile	0.051	(-0.0875, 0.1894)
Top Quartile	-0.053	(-0.1508, 0.0457)
*Difference*	*0.104*	*(-0.0715, 0.2786)*
By Internet Use:		
Non-Internet Users	0.081	(-0.1311, 0.2928)
Internet Users	-0.042	(-0.1005, 0.017)
*Difference*	*0.123*	*(-0.0993, 0.3445)*
By Observing Campaign News Online:		
No Campaign News Online	0.014	(-0.0773, 0.1045)
Campaign News Online	-0.048	(-0.1224, 0.0256)
*Difference*	*0.062*	*(-0.0505, 0.1745)*

Notes: Table shows the change between 2016 and 2012 (2016 minus 2012) in the weighted proportion of voting respondents that voted for the Republican presidential candidate, separately for groups that are more and less active online. We measure online activity using predicted internet use, actual internet use, and whether or not the respondent observed campaign news online. The difference row shows the difference in changes between the less active and more active group. The 95% confidence intervals are constructed via a nonparametric bootstrap at the respondent level with 100 replicates and taking the standard deviation of the statistic across replicates. See main text for details on variable construction and the SI Appendix of [5] for details on the nonparametric bootstrap procedure.

**Table 3:** Votes for Republican presidential candidate by alternative measures of predicted internet, 2012–2016.

Demographic group	Incremental *R*^2^	Change in proportion	95% CI
By Predicted Internet Use (Excludes age):			
Bottom Quartile	0.045	0.352	(0.2429, 0.4604)
Top Quartile		0.030	(-0.0756, 0.135)
*Difference*		*0.322*	*(0.1719, 0.472)*
By Predicted Internet Use (Excludes gender):			
Bottom Quartile	0.000	0.082	(-0.0412, 0.2052)
Top Quartile		-0.083	(-0.1724, 0.0069)
*Difference*		*0.165*	*(0.0262, 0.3033)*
By Predicted Internet Use (Excludes race):			
Bottom Quartile	0.015	0.050	(-0.0733, 0.1727)
Top Quartile		-0.049	(-0.1578, 0.0594)
*Difference*		*0.099*	*(-0.0749, 0.2727)*
By Predicted Internet Use (Excludes education):			
Bottom Quartile	0.115	-0.073	(-0.1895, 0.0438)
Top Quartile		-0.124	(-0.235, -0.0131)
*Difference*		*0.051*	*(-0.124, 0.2264)*
By Predicted Internet Use (Excludes south):			
Bottom Quartile	0.007	0.079	(-0.0422, 0.2)
Top Quartile		-0.090	(-0.1936, 0.0136)
*Difference*		*0.169*	*(-0.0049, 0.3428)*

Notes: Table shows the change between 2016 and 2012 (2016 minus 2012) in the weighted proportion of voting respondents that voted for the Republican presidential candidate, separately for groups that are more and less active online. Each measure re-constructs our main predicted internet measure after dropping separately each set of demographic covariates from the regression used to construct the predicted internet measure. The Incremental *R*^2^ is the the additive inverse of the change in *R*^2^ relative to the regression in Table 1. The difference row shows the difference in changes between the less active and more active group. The 95% confidence intervals are constructed via a nonparametric bootstrap at the respondent level with 100 replicates and taking the standard deviation of the statistic across replicates. See main text for details on variable construction and the SI Appendix of [5] for details on the nonparametric bootstrap procedure.

## Discussion

Relative to his predecessors, Trump performed worse among the demographic groups most likely to use the internet and social media. These facts do not rule out the possibility that these technologies advantaged Trump. It is possible that each of the three assumptions stated in the introduction could be violated:

1. It could be that content originating on social media was rebroadcast on traditional media such as cable television, and so persuaded non-internet users.
2. It could be that those least active online are those who are most influenced by internet content when exposed to it.
3. It could be that internet users would have been even less likely to vote for Trump absent new information technologies.

If social media content was a decisive factor in Trump’s victory, factors such as these must have been quantitatively significant. Until we have more precise quantitative evidence on the role of these factors, we think our findings caution against assuming that new technologies played a large role in the 2016 election outcome.

## Supporting information

S1 Appendix Model and supporting figure. (PDF)

S1 Replication Code (GZ)
