# Supplementary material for: A note on internet use and the 2016 U.S. presidential election outcome
Source: PLoS One. 2018 Jul 18;13(7):e0199571. doi: 10.1371/journal.pone.0199571 (PMC6051565; doi:10.1371/journal.pone.0199571)
Supplement: S1 Appendix — (PDF) [file pone.0199571.s001.pdf]

S1 Appendix:

A note on internet use and the 2016 U.S. presidential election outcome

Levi Boxell, *Stanford University*[*]
Matthew Gentzkow, *Stanford University and NBER*
Jesse M. Shapiro, *Brown University and NBER*

May 2018

[*]E-mail: lboxell@stanford.edu, gentzkow@stanford.edu, jesse_shapiro_1@brown.edu

1 Model

Let t index elections, with $t = 1$ denoting the 2016 election and $t = 0$ denoting some preceding election. Let r_t^g be the share of group g voting Republican in election t and let s_t^g be the share of the group that is internet active. Following [5], say that

$$\mathrm{E}\left(r_t^g | \alpha^g, s_t^g\right) = \alpha^g + \beta t + \rho_t s_t^g.$$

Under the assumptions stated in the introduction of the main text, the hypothesis that the internet advantaged the Republican candidate in 2016 is equivalent to $\rho_1 > \rho_0$, which implies that the Republican candidate performed better, on average, among internet-active groups in the 2016 election.

Appendix Figure 1: Trends in not voting or refusals by online activity.

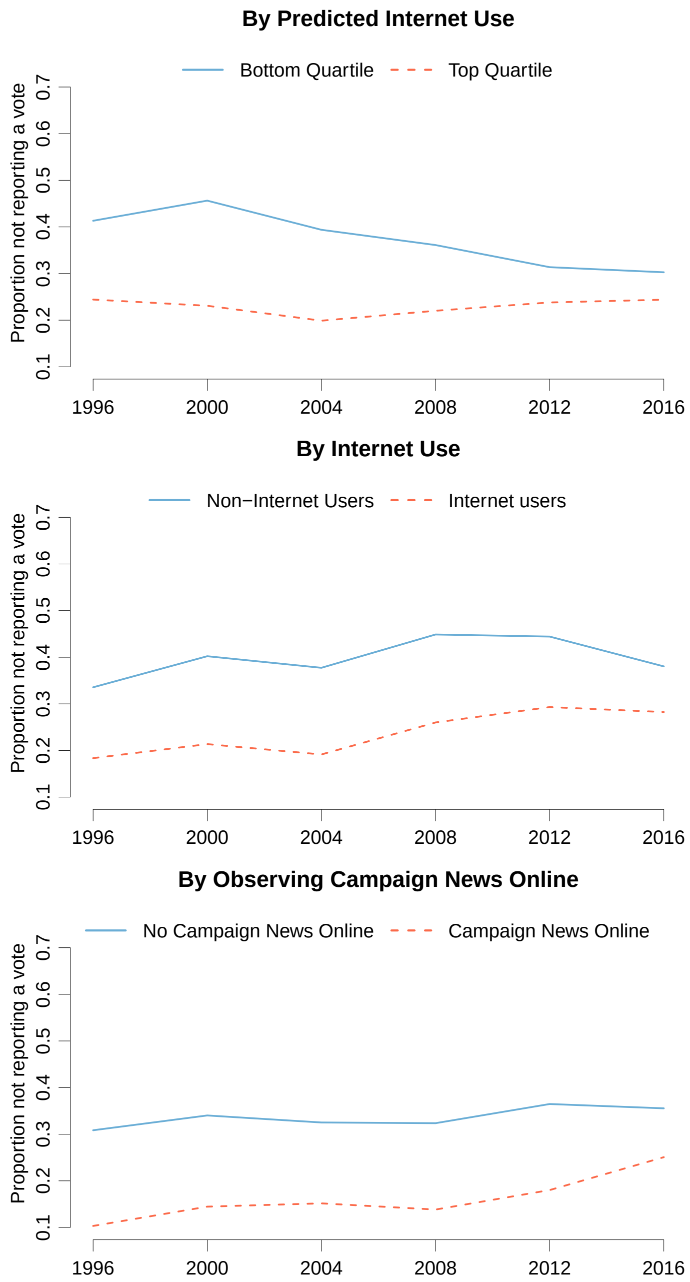

Notes: Plot shows trends in the weighted proportion of respondents that did not vote or refused to give a valid indication of their vote, separately for groups that are more and less active online. We measure online activity using predicted internet use, actual internet use, and whether or not the respondent observed campaign news online. See main text for details on variable construction.
