# Supplementary figures and images for: A note on internet use and the 2016 U.S. presidential election outcome
Source: PLoS One. 2018 Jul 18;13(7):e0199571. doi: 10.1371/journal.pone.0199571 (PMC6051565; doi:10.1371/journal.pone.0199571)

## By Predicted Internet Use

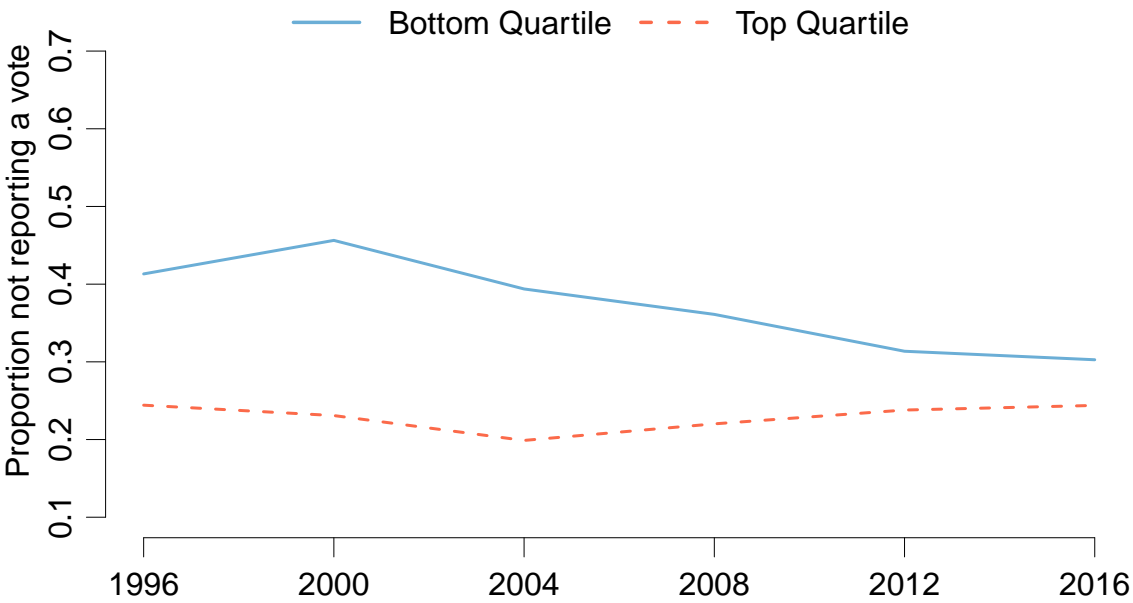

## By Internet Use

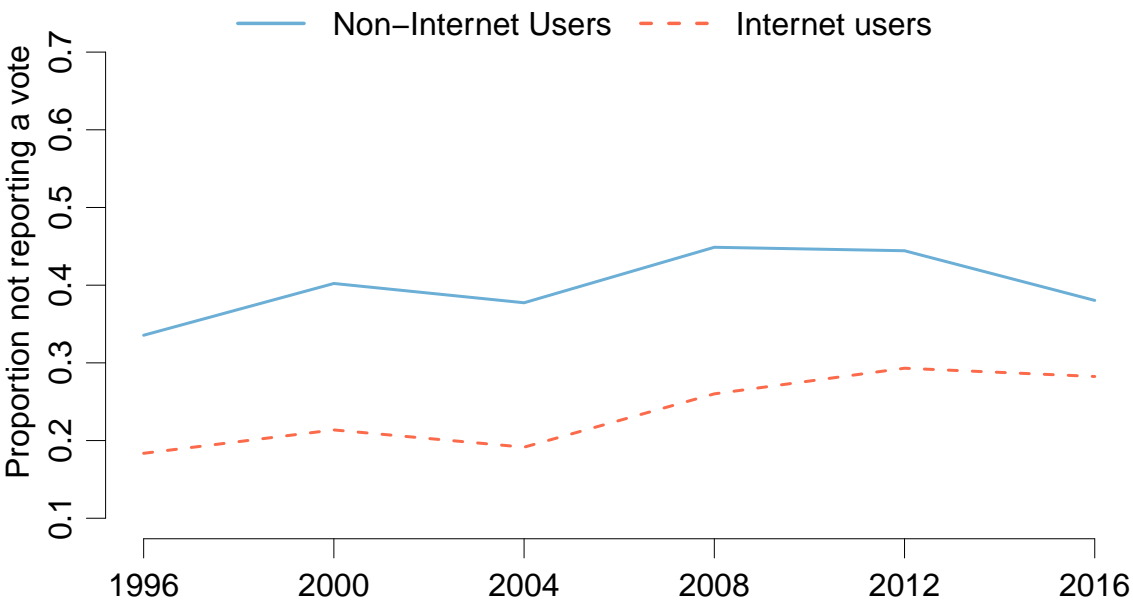

## By Observing Campaign News Online

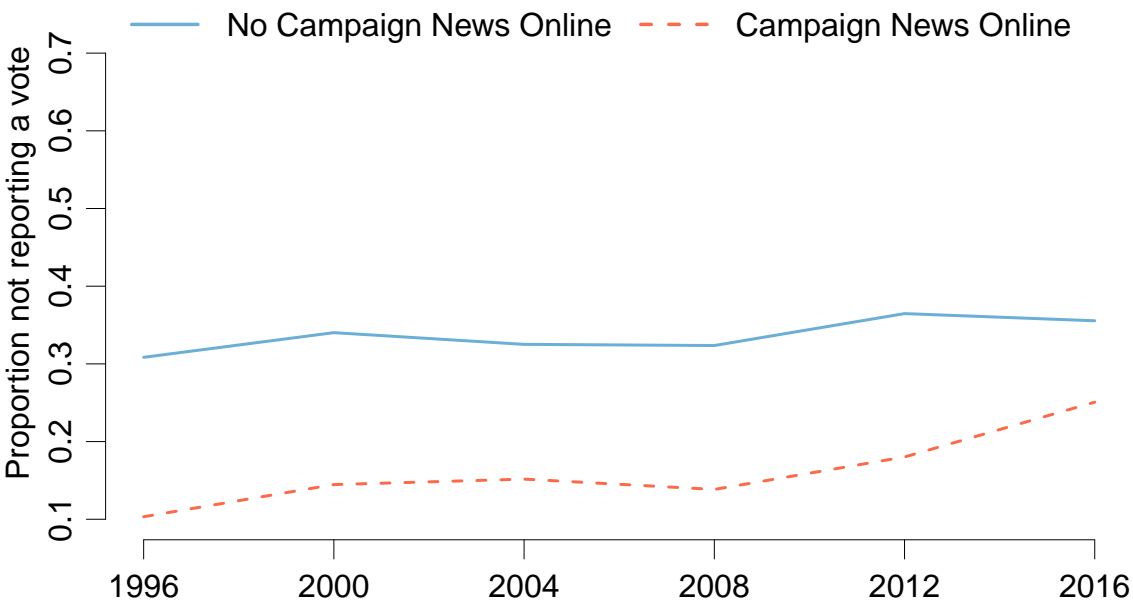

Supplement: S1 Replication Code — (GZ) [file pone.0199571.s002.tar.gz › Archive/release/analysis/plots_and_tables/nonvote_by_internet.pdf]

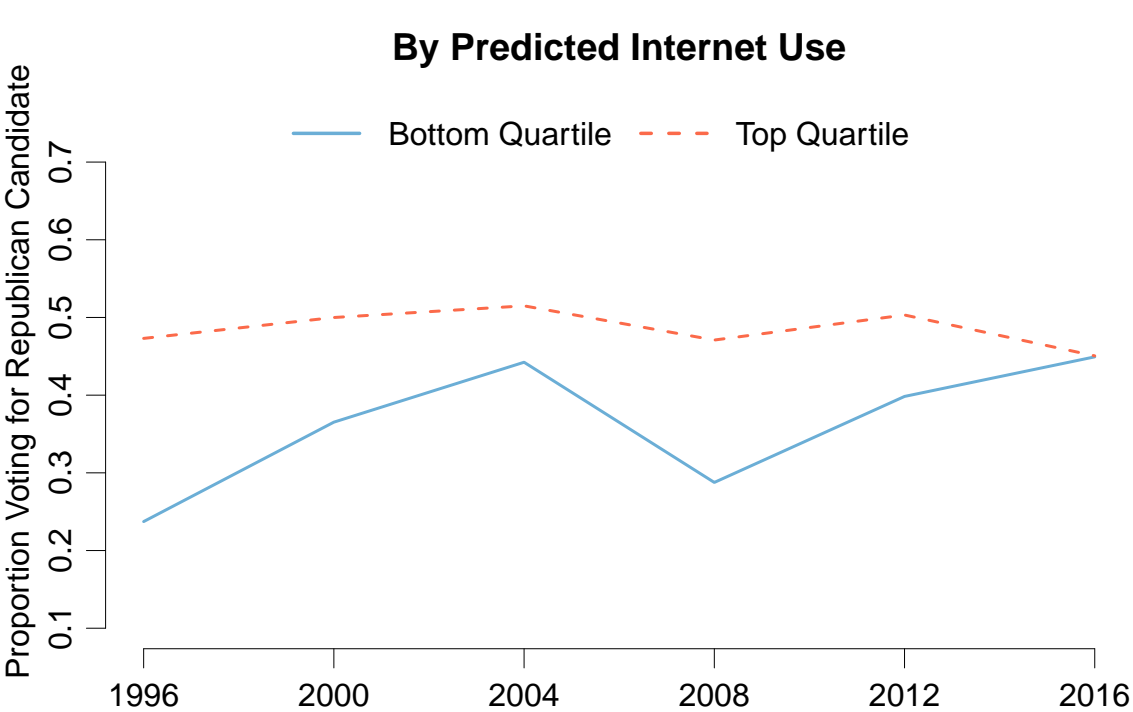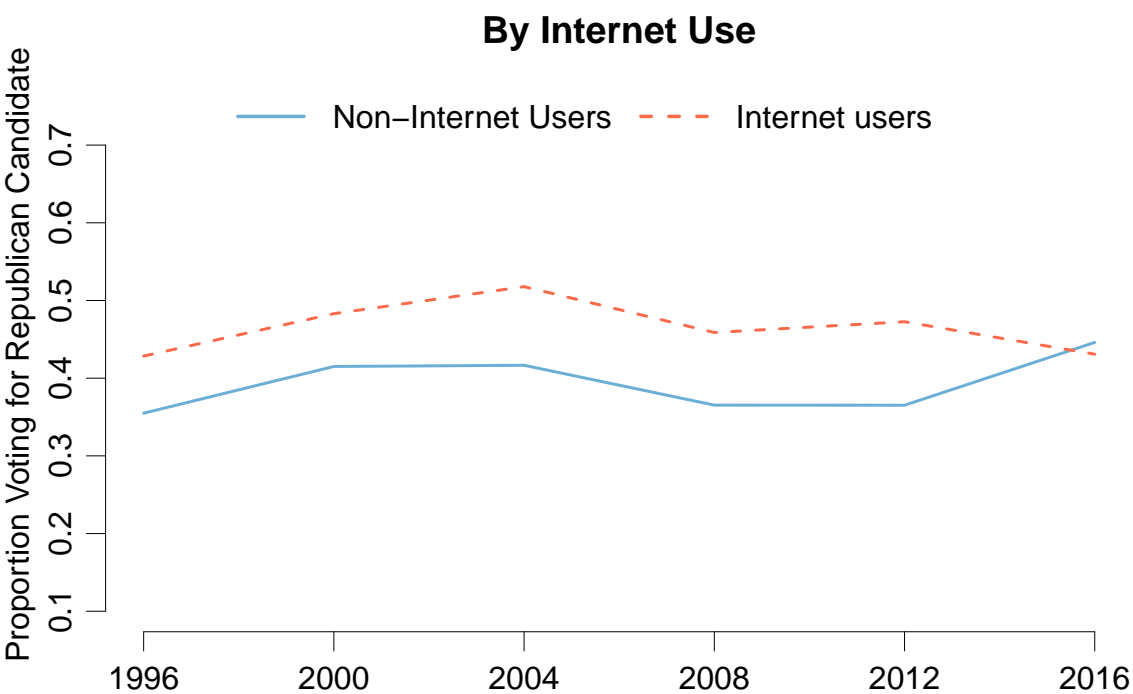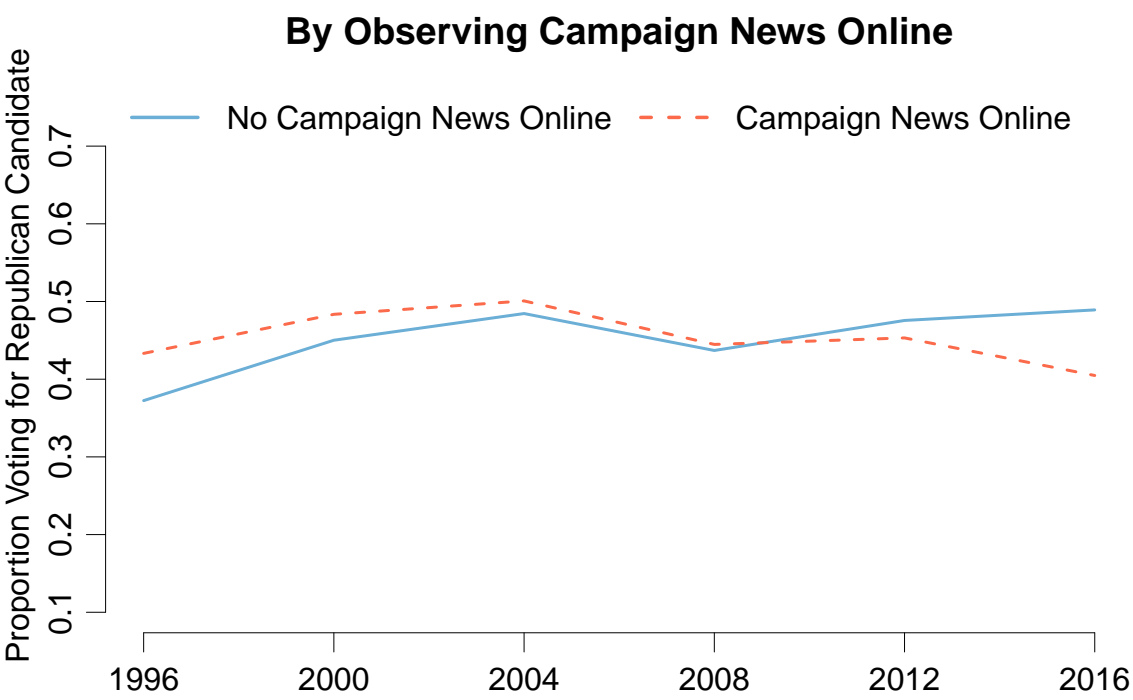

Supplement: S1 Replication Code — (GZ) [file pone.0199571.s002.tar.gz › Archive/release/analysis/plots_and_tables/republican_voteshare_by_internet.pdf]
